# The cost and cost drivers of delivering COVID-19 vaccines in low- and middle-income countries: a bottom-up costing study of rollouts in seven countries

**DOI:** 10.1371/journal.pone.0341964

**Published:** 2026-02-02

**Authors:** Flavia Moi, Văn Minh Nguyễn, Rachel Archer, Tozé Namalela, Christina Banks, Tarek Hossain, Afroja Yesmin, Cathbert Tumusiime, Charlotte Muheki, Kelsey Vaughan, Elise Smith, Rafael Deo Estanislao, Pierre Z. Akilimali, Hong Thi Duong, Chien Chinh Vien, Amélia Dipuve, Pedro Marizane Pota, Monjurul Islam, Paul Kiggundu, Okello Ayen Daniel, Sarah De Los Reyes, Jeremie de Guzman, Christelle Cuevas, Primrose Nakazibwe, Carl Schutte, Minh Van Hoang, Laura Boonstoppel

**Affiliations:** 1 ThinkWell, Geneva, Switzerland; 2 Hanoi University of Public Health, Hanoi, Vietnam; 3 ThinkWell, Manchester, United Kingdom; 4 ThinkWell, Maputo, Mozambique; 5 ThinkWell, Boston, Massachusetts, United States of America; 6 ThinkWell, Dhaka, Bangladesh; 7 ThinkWell, Kampala, Uganda; 8 Genesis Analytics, Johannesburg, South Africa; 9 ThinkWell, Manila, The Philippines; 10 Patrick Kayembe Research Center, Kinshasa School of Public Health, University of Kinshasa, Kinshasa, the Democratic Republic of the Congo; 11 National Institute of Hygiene and Epidemiology, Hanoi, Vietnam; 12 Tay Nguyen Institute of Hygiene and Epidemiology, Dak Lak, Vietnam; 13 Expanded Program on Immunization, Mozambique Ministry of Health, Maputo, Mozambique; 14 Centro de Estudos de Economia e Gestão, Universidade Eduardo Mondlane, Maputo, Mozambique; 15 Expanded Programme on Immunization (EPI), Directorate General of Health Services, Dhaka, Bangladesh; 16 Kampala Capital City Authority, Kampala, Uganda; 17 Ateneo de Manila School of Medicine and Public Health, Manila, The Philippines; 18 Ateneo de Manila University, Manila, The Philippines; 19 Ndejje University, Kampala, Uganda; University of Waterloo, CANADA

## Abstract

**Introduction:**

While COVID-19 vaccines were crucial in containing the pandemic, there is limited evidence on the cost of delivering them in low- and middle-income countries. We estimated the cost of delivering COVID-19 vaccines in Bangladesh, Côte d’Ivoire, the Democratic Republic of the Congo, Mozambique, the Philippines, Uganda, and Vietnam.

**Methods:**

We retrospectively estimated the financial and economic cost of COVID-19 vaccine delivery from a payer perspective, using a bottom-up approach. Cost data were collected from 290 sites, while qualitative interviews were conducted with 192 key informants. We estimated volume-weighted average costs per dose in 2022 USD, for introduction phases, delivery modalities, and strategies.

**Results:**

The financial cost per dose ranged from $0·29-$2·18 across countries, driven by per diem and supplies. The economic cost per dose ranged from $1·14-$9·50, driven by the cost of labor. Newly hired health workers were a cost driver only in the Philippines. The economic delivery cost per dose was inversely correlated with daily vaccine volume delivered at vaccination sites. Similarly, delivering through campaigns came at a lower unit cost than continuous delivery, and when programs scaled up, the cost per dose decreased dramatically. Political prioritization, health workers’ commitment, and volunteers were highlighted as success factors, while resource constraints at implementation level and health workers shortages were flagged as key challenges.

**Conclusion:**

Our findings demonstrate how under-resourced health systems managed to deliver massive amounts of vaccines with relatively few resources. However, they also expose significant gaps and inefficiencies, and underscores the need to invest in resilient health systems to improve future pandemic responses.

## Introduction

The coronavirus disease 2019 (COVID-19) quickly spread across the world and was declared a Public Health Emergency of International Concern by the World Health Organization (WHO) in January 2020 [1,2]. By the time the WHO declared the end of COVID-19’s global health emergency in May 2023, the disease had caused almost 7 million deaths globally, overwhelming health systems and significantly affecting access to and delivery of essential health services [3–5].

Immediately recognized as a crucial tool in containing the disease, COVID-19 vaccines were developed at an unprecedented speed and were introduced less than a year later, in December 2020. Initial supply constraints meant that vaccines had to be rolled out in a phased manner, initially only targeting high-risk individuals. As production ramped up, countries quickly expanded their target population to encompass all adults, with some even including children. A year after they first hit the market, nearly every country in the world had introduced COVID-19 vaccines. By the end of 2021, 9·23 billion doses had been administered worldwide, making it the fastest and largest vaccine rollout in history [6].

Recognizing the need for equitable access to an initially limited supply, the international community established the COVID-19 Vaccines Global Access initiative (COVAX) with the goal of accelerating access to COVID-19 vaccines in low- and middle-income countries (LMICs) [7]. Although all 92 COVAX-supported LMICs introduced COVID-19 vaccines in 2021, vaccine introductions were fraught with many financial and operational challenges. Initially, pre-purchase agreements and export bans by high-income countries delayed introductions in LMICs, resulting in an intermittent vaccine supply that was challenging to distribute. Later, already under-resourced health systems struggled to effectively deliver the large volumes required to reach WHO’s recommended 70% population coverage, and operational support from partners and donors to fill gaps was limited and often delayed. Some vaccines required ultra-cold chain, which many LMICs did not have, and many programs struggled with vaccine hesitancy [8].

Despite the unique characteristics of the COVID-19 vaccine rollout, there is limited evidence on the cost of delivering COVID-19 vaccines, especially from LMICs. Nine studies have been published on the cost of delivering COVID-19 vaccines in LMICs [9–17]. Most contain modelled estimates based on the cost of other vaccination programs, broad-level assumptions, or rollout plans that did not reflect actual practices, and few are based on actual resource use at implementation level. Moreover, comparability is limited by the different methodologies used in the studies.

Understanding the cost and cost drivers of COVID-19 vaccine introductions, and how these varied across LMICs, is essential to improve pandemic preparedness, outbreak responses, and integration of adult vaccination programs into primary health care. To fill this evidence gap, we conducted bottom-up studies on the cost of delivering COVID-19 vaccines in seven LMICs.

## Methods

### Study design

This study retrospectively estimated the costs of delivering COVID-19 vaccines, using a bottom-up, ingredients-based approach, in seven purposively selected low- and middle-income countries: Bangladesh, the Democratic Republic of the Congo (DRC), Côte d’Ivoire, Mozambique, the Philippines, Uganda, and Vietnam. Country-specific protocols followed a standardized methodology to enhance the comparability of results, and received ethical approval in each of the countries. Specifically, ethical approval was granted by the following institutions: the Institutional Review Board of the Institute of Health Economics in Bangladesh, the Comité National d’Éthique des Sciences de la Vie et de la Santé in Côte d’Ivoire, the Ethical Committee of the School of Public Health of the University of Kinshasa in the DRC, the Universidade Eduardo Mondlane’s Faculty of Medicine and Maputo Central Hospital joint ethical review board in Mozambique, the Single Joint Research Ethics Board (SJREB) of the Department of Health (DOH) in the Philippines, the Mildmay Uganda Research & Ethics Committee in Uganda, the Institutional Review Board at Hanoi University of Public Health in Vietnam [18]. The study was conducted from a payer perspective including costs incurred at all levels of the health system by health service providers, ministries of health (MOH), national vaccine cold stores, and development partners. In Bangladesh, costs were also estimated from a beneficiary perspective, capturing costs incurred by individuals to receive a single vaccine dose, and those findings can be found in a separate publication [19]. In all countries, we also conducted qualitative interviews to understand how COVID-19 vaccination programs operated and were financed.

### Study setting and context

All seven countries introduced COVID-19 vaccines between January and April 2021, but their vaccination programs varied significantly in terms of delivery strategies, speed of uptake, volume delivered, and coverage (Table 1). Bangladesh, the Philippines, and Vietnam delivered many more vaccines compared to the African countries, due to high coverage achieved among much larger target populations. Mozambique and Uganda also achieved high vaccination coverage, though with lower daily delivery volumes. Côte d’Ivoire and the DRC struggled with low uptake at the time of this study, and while in Côte d’Ivoire coverage later picked up thanks to additional partner support, by December 2022 the DRC had delivered less than ten doses per 100 people, due to high vaccine hesitancy and operational bottlenecks [6].

**Table 1:** Overview of the COVID-19 vaccination programs.

	Cumulative COVID-19 doses delivered[6]				Delivery modality	Delivery strategies
	By December 2021		By December 2022			
	In millions	Per 100 peoplea	In millions	Per 100 peoplea		
**Bangladesh**	133·1	78·6	334·8	197·7	Continuous and campaigns	Fixed and temporary sites
**Côte d’Ivoire**	7·1	23·4	23·8	78·2	Continuous with monthly intensification periods (10-15 days)	Fixed and temporary sites, outreach/mobile, vaccinodromes
**The DRC**	0·3	0·3	10·0	9·8	Continuous and campaigns	Fixed and temporary sites, mobile teams, vaccinodromes
**Mozambique**	15·7	45·1	29·1	89·2	Continuous	Fixed and temporary sites, mobile teams
**The Philippines**	108·5	95·2	169·8	149·0	Continuous and campaigns	Fixed and temporary sites, mobile
**Uganda**	12·1	25·5	26·3	55·6	Continuous	Fixed and temporary sites, and outreach
**Vietnam**	150·9	151·4	265·5	266·3	Round-based	Fixed and temporary sites

^a^In some countries, estimates exceed 100 doses delivered per 100 people because of second doses and booster doses.

Most countries delivered vaccines continuously, and some complemented this with periods of intensified delivery or campaigns. Vietnam organized vaccination in rounds, each corresponding to a new vaccine shipment’s arrival. Countries delivered vaccines at health facilities and at temporary vaccination sites in the community—including offices, community centers, malls, schools, and others. Côte d’Ivoire, the DRC, Mozambique, the Philippines and Uganda also conducted outreach or mobile delivery or set up vaccinodromes (large temporary sites planned to deliver high volumes of vaccines).

COVID-19 vaccination programs in all countries were financed by a mix of government and donor funding. Most vaccines were supplied through COVAX. All countries also received bilateral donations or self-procured. Donors and partners provided technical assistance and also supported activities such as social mobilization, training, and vaccine distribution.

### Scope

We defined delivery costs as all costs borne by the health system, providers, donors and implementing partners associated with vaccinating the target population, excluding the cost of vaccine procurement. We included financial costs—financial outlays specifically related to the COVID-19 vaccination program—as well as economic costs—defined as the sum of financial costs and opportunity costs, the latter representing the value of using existing resources, primarily labor and annualized and discounted capital costs. Resource types and program activities included are defined in S1 and S2 Tables. In each country, we collected recurrent costs for a period of one to four months between March 2021 and July 2022, as well as one-off investments made for the COVID-19 vaccine program ahead of the rollout until the end of the recurrent costs period. The precise data collection period per country can be found in S3 Table. Depending on data availability, we disaggregated findings by introduction phase (initial rollout targeting priority groups vs scaled up phase targeting general population), delivery modality (continuous vs campaigns), and delivery strategy or type of delivery site. Table 2 illustrates for what country each disaggregated analysis was conducted and explains why the disaggregation was not possible in some countries.

**Table 2:** Overview of disaggregated analysis conducted for each country.

	Introduction phase	Delivery modality	Delivery strategy or type of site
**Bangladesh**	No, data not collected for initial rollout	Yes	Yes
**Côte d’Ivoire**	No, due to data limitations	No, due to data limitations	No, due to data limitations
**The DRC**	No, data not collected for initial rollout	Yes	No, due to data limitations
**Mozambique**	Yes	Not applicable	No, due to data limitations
**The Philippines**	No, data not collected for initial rollout	Yes	Yes
**Uganda (Kampala)**	Yes	Not applicable	No, due to data limitations
**Vietnam**	Yes	Not applicable	Yes

### Data collection & sampling

In each country we consulted with the MOH to select a sample, with the exception of Uganda, where we worked with the Kampala Capital City Authority, a semi-autonomous body that defines and plans its own immunization program. Purposive sampling was conducted to rapidly select sites. We selected a mix of rural and urban areas, high- and low-volume sites, and various delivery strategies. Non-governmental partners contributing to the vaccination effort were also identified and included. The full sample is described in Table 3 below.

**Table 3:** Study sample, by level of the health system and for partner organizations.

Cost data	National level offices	Intermediary administrative sites (regions, cities, provinces, districts, health zones)	Implementation sites (health facilities and other vaccination sites)	Partners	Total
**Bangladesh**	1	0	38	2	**41**
**Côte d’Ivoire**	3	9	30	5	**47**
**The DRC**	1	20	26	4	**51**
**Mozambique**	1	8	27	5	**41**
**The Philippines**	1	9	27	0	**37**
**Uganda**	0^a^	6	28	1	**35**
**Vietnam**	1	9	26	2	**38**
**Total**	**8**	**61**	**202**	**19**	**290**

^a^The Uganda study focused only on Kampala City

Data were collected through in-person interviews, complemented with the review of financial reports, health facility registers, and other written records which were accessed and reviewed on site. Cost data were collected from a total of 290 implementation sites, administrative offices, and partners, using a standardized Excel-based data collection tool. Qualitative data were collected from all administrative and a subset of implementation sites, through 192 interviews performed using semi-structured questionnaires. All respondents provided written informed consent. Data collection cost and qualitative interviews were conducted in all countries between April 2022 and July 2023, generally one to three months after the period for which recurrent costs were collected (listed in the S3 Table). All data sheets were anonymized before analysis. Missing data were imputed following the methods described in S4 Table.

### Data analysis

Costs were estimated by multiplying the quantity of resources used by their price or value. Shared costs were allocated to the COVID-19 vaccination program and activities as per the rules described in S5 Table. All costs were first inflated to 2022 in the local currency, using the Consumer Price Index published by the International Monetary Fund, and then converted into United States dollars (USD, $). Capital costs and one-off investments were annualized. Depreciation costs were estimated using replacement prices and standard useful life assumptions, and a discount rate of 3% [20]. For each level, we estimated the volume-weighted average cost per dose (i.e., weighing the average cost per dose by the number of doses delivered at each site) and summed these to estimate the overall cost per dose. We performed a thematic analysis of the qualitative interviews to identify common themes. First, a country-specific qualitative analysis was conducted by at least two investigators. The depth of analysis varied across countries, depending on the availability of interview transcripts. In Uganda and Bangladesh, interview transcripts in the original language were thematically analyzed through systematic coding to identify key themes. In Vietnam, interviews were recorded, transcribed, and synthesized in Vietnamese prior to translation into English. In the Philippines, the Democratic Republic of the Congo, Mozambique, and Côte d’Ivoire, detailed interview notes were translated into English and summarized to identify patterns, lessons learned, and insights related to program implementation. Upon completion of the country-level analyses, two investigators conducted a cross-country thematic synthesis, systematically reviewing and refining themes to capture commonalities and contrasts across contexts.

## Results

### Enablers and challenges

Our thematic analysis found that key factors that enabled the success of COVID-19 vaccination programs included political prioritization, exceptional commitment from health workers, and a massive mobilization of all available resources—including recruitment of volunteers, redeployment of health workers from other health facilities, and, in the Philippines, mass hiring of health workers—as illustrated in Table 4.

**Table 4:** Enablers and challenges in the implementation of COVID-19 vaccination programs.

	Enablers	Challenges
**Leadership and management**	Exceptional political commitment and oversight *[Ban, Moz, Uga, Phil]* Effective coordination across administrative levels or government entities *[CdI, Ban, Phil, Uga]*	None
**Financing**	Support from donors and development partners for vaccine procurement and operational cost *[All]* Tailored financing mechanisms to ensure timely disbursement of donor funding to vaccination sites *[Ban]*	Insufficient funding at implementation level, particularly for transport and incentives *[All]* Delays in disbursements due to cumbersome public financial management regulations *[Ban, Moz, Phil]*
**Human resources**	Extraordinary commitment from health workers to reach vaccination targets *[All]* Mass hiring of additional health workers *[Phil]* Redeployment of staff from lower-priority health facilities or other geographic areas and sectors to boost capacity where it was most needed *[Phil, Viet, Uga]* Mass mobilization of volunteers *[Viet, Ban, Moz, Uga, DRC, CdI]*	Overworked health workforce due to high pressure and workload, and limited ability to fill (pre-existing) human resource shortages *[Viet, Ban, Uga, DRC, CdI, Moz]* Low morale among staff and volunteers due to insufficient and unpaid allowances *[CdI, Ban, Moz, Uga, DRC, Viet]* Health workers sometimes had to contribute their own resources to make up for shortages (e.g., personal vehicles, fuel, etc.) *[Ban, DRC, Moz, CdI, Phil]*
**Community mobilization**	High degree of trust in the Expanded Programme on Immunization (EPI) ensured high uptake of COVID-19 vaccines *[Ban]* Local community committees provided support for coordination and social mobilization, and informed outreach strategies *[Viet, Uga, Phil]*	Severe vaccine hesitancy *[DRC]*
**Training**	Leveraging of virtual technologies to train health workers during the pandemic *[Ban, Moz, Viet, DRC, Uga, Phil]*	Multitude of trainings held as new vaccine products were added considered burdensome by health workers [*Viet*]
**Recordkeeping and reporting**	None	Challenges in the rollout of COVID-19 vaccine reporting software led to increased workload for health workers or lack of facility-level doses-delivered data *[Viet, Moz]* Inconsistent, fragmented recordkeeping and untimely reporting hindered data quality and availability for decision-making *[Uga, Phil]*
**Cold chain equipment and vaccine distribution**	Non-health private sector companies supported vaccine distribution and offered space for vaccination sites *[Ban, Viet, Uga]*	Supply constraints when the vaccine program was first introduced resulting in costly, small delivery batches *[All]* Insufficient cold chain equipment at implementation level increased frequency of trips to higher levels or nearby facilities *[DRC, Ban, Viet, Phil]*

Abbreviations: Ban = Bangladesh; CdI = Côte d’Ivoire; DRC = the Democratic Republic of the Congo; Moz = Mozambique; Phil = the Philippines; Viet = Vietnam; Uga = Uganda

Key challenges raised by informants mainly related to shortages in staffing, funding, cold chain equipment, and transport. Despite financial support from donors, financial resources for COVID-19 vaccination programs were perceived as insufficient, particularly at implementation level. Additionally, several countries reported funding delays, due to complex domestic public financial management regulations, and untimely disbursements from donors. Only in Bangladesh tailored financing mechanisms were put in place to expedite disbursements of donor support to ensure timely availability at implementation level. Finally, supply constraints and insufficient cold chain equipment at implementation level were identified as bottlenecks that drove up transport cost, as vaccines had to be picked up frequently in small batches.

### Descriptive statistics

The average COVID-19 vaccine volume delivered varied from 35 doses per site per day in the DRC to 543 in Bangladesh (see Table 5). Most countries did not hire (Bangladesh, Mozambique, Vietnam), or hired very few additional health staff for COVID-19 vaccination (Côte d’Ivoire, DRC, Uganda), due to very limited or not existing spare capacity in the health workforce of these countries. Mass hiring of health workers was only observed in the Philippines. Some countries redeployed existing staff from other areas (Vietnam, the Philippines, and Uganda). Volunteers were leveraged in all countries.

**Table 5:** Description of the sample.

	Bangladesh	Côte d’Ivoire	The DRC	Mozambique	The Philippines	Uganda	Vietnam
**Sampled implementation sites**	38	30	26	27	27	28	26
**COVID-19 vaccine doses delivered per site/day (Q1-Q3)** ^**a**^	543 (191-667)	55 (8-80)	35 (8-49)	156 (17-401)	370 (208-525)	120 (41-133)	189 (68-239)
**Average number of COVID-19 vaccine staff at implementation sites**	28	11	5	10	35	22	19
Existing health staff^b^ (%)	64%	45%^d^	71%	49%	73%	67%^d^	77%
Volunteers & unpaid health staff (%)	36%	45%^d^	28%	51%	1%	31%^d^	23%
Newly hired health staff (%)	0%	9%^d^	1%	0%	26%	3%^d^	0%
**% sites with additional financial incentives**^**c**^ **for COVID-19 vaccine delivery**	95%	33%	43%	19%	78%	75%	100%
**% sites with new cold chain equipment to support the COVID-19 vaccination program**	18%	0%	4%	0%	52%	0%	8%

^a^Q1: 25^th^ percentile; Q3: 75^th^ percentile.

^b^Includes redeployed staff.

^c^Includes per diem, incentives, volunteer allowance and refreshments.

^d^Does not add up to 100% due to rounding error.

Most health workers did not receive additional incentives for the COVID-19 vaccination program. Vietnam was the only country where health workers consistently received an incentive. In all other countries, incentives were only given for very short periods of time, or only for specific activities like campaigns, or only to volunteers. Very few sites reported any additional investments in cold chain equipment, except in the Philippines and some in Bangladesh.

### Delivery cost and cost drivers across countries

The financial delivery cost per dose, exclusive of vaccine procurement, ranged from $0·29 to $2·18, and the economic cost per dose ranged from $1·05 to $9·50 across countries, as shown in Table 6. While the cost per dose was lowest in Bangladesh, where delivery volume was highest, financial and economic costs per site per day were lowest in Côte d’Ivoire where the volume delivered was considerably lower.

**Table 6:** Cost per dose, cost per site per day, and doses delivered per site per day.

	Bangladesh	Côte d’Ivoire	The DRC	Mozambique	The Philippines	Uganda	Vietnam
**Cost and volume delivered per site/day**							
Doses delivered per site/day	543	55	35	156	370	120	189
Financial cost per site/day	$160	$37	$76	$78	$736	$98	$113
Economic cost per site/day	$570	$174	$333	$178	$1,295	$292	$336
**Financial cost per dose in 2022 USD** (% of economic costs per dose)							
Labor – newly hired health staff	$0·0	$0·02	$0·03	··	$1·11	$0·08	$0·001
Per diem & incentives^a^	$0·20	$0·10	$0·77	$0·04	$0·13	$0·24	$0·27
Vaccine admin. supplies	$0·04	$0·37	$0·51	$0·19	$0·23	$0·25	$0·20
Transport and fuel	$0·02	$0·04	$0·23	$0·18	$0·05	$0·08	$0·03
Other financial costs^b^	$0·03	$0·03	$0·64	$0·09	$0·42	$0·16	$0·09
New equipment (e.g., cold chain, vehicles)	$0·01	$0·10	$0·002	$0·00	$0·05	$0·01	$0·01
**Total financial cost per dose**	**$0**·**29** **(28%)**	**$0**·**67 (21%)**	**$2**·**18 (23%)**	**$0**·**50 (44%)**	**$1**·**99 (57%)**	**$0**·**82 (34%)**	**$0**·**60 (34%)**
**Opportunity cost per dose in 2022 USD** (% of economic costs per dose)							
Labor – existing health staff	$0·66	$2·21	$3·93	$0·48	$1·41	$1·13	$0·97
Unpaid labor	$0·07	$0·27	$2·97	$0·07	$0·03	$0·19	$0·18
Other opportunity costs	$0·02	$0·01	$0·42	$0·09	$0·07	$0·29	$0·03
**Total opp. cost per dose**	**$0**·**75** **(72%)**	**$2**·**48 (79%)**	**$7**·**33 (77%)**	**$0**·**64 (56%)**	**$1**·**51 (43%)**	**$1**·**61 (66%)**	**$1**·**18 (66%)**
**Economic cost per dose**	**$1**·**05**	**$3**·**16**	**$9**·**50**	**$1**·**14**	**$3**·**50**	**$2**·**43**	**$1**·**78**

^a^Also includes refreshments for vaccination team members and allowances for volunteers; ^b^ For DRC, other costs are mostly made up of donor contributions spent on unknown resource types

In Vietnam, Bangladesh, and Uganda, per diem and incentives for vaccination team members were a financial cost driver. Vaccine safety and administration supplies were an important cost driver in Côte d’Ivoire, Uganda, and Vietnam. Labor for newly hired staff was a financial cost driver only in the Philippines. Costs related to investments in new equipment were negligible in all countries except Côte d’Ivoire. Opportunity costs represented 43% to 79% of the economic cost per dose, and mostly consisted of staff salaries, and in some countries, the value of unpaid labor.

Delivery costs disaggregated by program activity, and by delivery strategy or sites can be found in S6, S7, and S8 Tables.

### Delivery cost during different introduction phases

In Mozambique, Uganda, and Vietnam, the delivery cost per dose, exclusive of vaccine procurement, was significantly higher during the initial rollout—when countries were targeting only priority populations and dealing with supply constraints—compared to when COVID-19 vaccination programs were delivering at scale, as shown in Table 7. This meant that although the number of doses delivered per site per day was much greater when the program delivered at scale, the cost per site per day increased only a little or even decreased.

**Table 7:** Cost per dose, total costs and doses delivered per site per day, for different introduction phases.

	Mozambique		Uganda		Vietnam	
Introduction phase	Initial rollout	At scale	Initial rollout	At scale	Initial rollout	At scale
**Cost and volume delivered per site/day**						
Number of sampled sites that were active	20	27	20	28	16	23
Dose delivered per site/day	64	225	89	149	77	267
Financial cost per site/day	$62	$97	$134	$98	$163	$155
Economic cost per site/day	$229	$191	$386	$294	$413	$451
**Financial cost per dose in 2022 USD** (% of economic costs per dose)						
Labor – newly hired health staff	··	··	$0·16	$0·06	$0·01	$0·00
Per diem & incentives^a^	$0·10	$0·03	$0·51	$0·17	$0·79	$0·26
Vaccine admin. supplies	$0·32	$0·16	$0·30	$0·24	$0·21	$0·21
Transport and fuel	$0·10	$0·16	$0·17	$0·06	$0·62	$0·02
Other financial costs	$0·44	$0·07	$0·35	$0·11	$0·38	$0·09
New equipment (e.g., cold chain, vehicles)	$0·00	$0·00	$0·03	$0·01	$0·12	$0·00
**Total financial cost per dose**	**$0**·**96 (27%)**	**$0**·**43 (51%)**	**$1**·**51 (35%)**	**$0**·**66 (34%)**	**$2**·**12 (40%)**	**$0**·**58 (34%)**
**Opportunity cost per dose in 2022 USD** (% of economic costs per dose)						
Labor – existing health staff	$2·27	$0·31	$1·99	$0·92	$2·82	$0·92
Unpaid labor	$0·24	$0·05	$0·35	$0·15	$0·00	$0·18
Other opportunity costs	$0·08	$0·06	$0·48	$0·24	$0·42	$0·02
**Total opportunity cost per dose**	**$2**·**59 (73%)**	**$0**·**42 (49%)**	**$2**·**82 (65%)**	**$1**·**31 (66%)**	**$3**·**24 (60%)**	**$1**·**11 (66%)**
**Economic cost per dose**	**$3**·**56**	**$0**·**85**	**$4**·**34**	**$1**·**97**	**$5**·**36**	**$1**·**69**

^a^Also includes refreshments for vaccination team members and allowances for volunteers

### Delivery cost for continuous and campaign delivery modalities

Per diem and incentives were given more frequently for campaigns than for continuous delivery, and the daily amount given to each worker was also higher (see Table 8). In the DRC and the Philippines, campaigns were associated with higher volumes delivered at lower unit costs. In Bangladesh, delivery volumes were lower during campaigns and the associated cost per dose was higher, because the campaigns we observed there were implemented when the country had already reached high vaccination coverage. S9 Table provides a breakdown of the cost per dose by resource type for continuous and campaign delivery.

**Table 8:** Cost per dose, total cost and doses delivered per site per day, for different delivery modalities.

	Bangladesh		The DRC		The Philippines	
Delivery modality	Continuous	Campaign	Continuous	Campaign	Continuous	Campaign
Number of sampled sites that were active	26	6	26	26	26	24
% of sites with per diem^a^	92%	100%	23%	35%	42%	71%
Average daily per diem^a^ per staff (at sites where they were provided)	$2·95	$5·24	$4·78	$10·46	$1·90	$3·54
**Cost and delivery volume per site/day**						
Average number of dose delivered per site/day	493	320	18	52	161	598
Financial cost per site/day	$148	$106	$56	$99	$336	$665
Economic cost per site/day	$523	$346	$352	$317	$597	$1,018
**Cost per COVID-19 vaccine dose delivered in 2022 USD** (% of the economic cost per dose)						
Financial cost per dose	$0·30 (28%)	$0·33 (31%)	$3·12 (16%)	$1·91 (31%)	$2·35 (55%)	$1·41 (65%)
Opportunity cost per dose	$0·76 (72%)	$0·75 (70%)	$16·45 (84%)	$4·19 (69%)	$1·95 (45%)	$0·75 (35%)
**Economic cost per dose**	**$1**·**06**	**$1**·**08**	**$19**·**56**	**$6**·**10**	**$4**·**29**	**$2**·**16**

^a^Also includes refreshments for vaccination team members and allowances for volunteers

### Relationship between delivery cost per dose at sites and volume delivered

**Fig 1:**
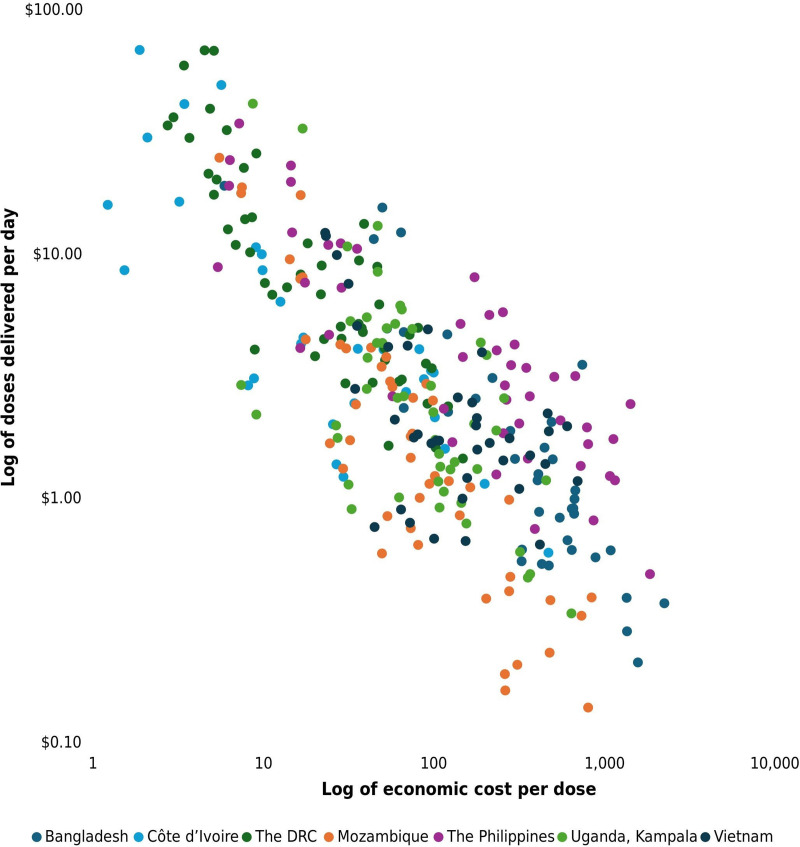
Relationship between the economic delivery cost per dose at implementation sites and volume delivered per day at sites (n = 300).

We found an inverse power-law relationship between the economic delivery cost per dose incurred at implementation sites, exclusive of vaccine procurement, and the volume delivered per day, as illustrated in Fig 1. We did not find this relationship when looking at financial costs, because most of the financial costs were for resources that scale linearly with volume delivered.

## Discussion

This study found that the financial cost per dose, exclusive of vaccine procurement, for delivering COVID-19 vaccines in the first year and a half of their introduction ranged from $0·29 in Bangladesh to $0·50 in Mozambique, $0·60 in Vietnam, $0·67 in Côte d’Ivoire, $0·82 in Uganda, $1·99 in the Philippines, and $2·18 in the DRC. Financial cost drivers included per diem and incentives (in Bangladesh, Vietnam, and Uganda), vaccine administration supplies (in Côte d’Ivoire, Mozambique, Uganda, and Vietnam), and newly hired health workers (in the Philippines). The economic cost per dose found in our study—which ranged widely from $1·14 in Mozambique to $9·50 in the DRC—was driven by the cost of paid labor and value of unpaid labor. Differences in delivery cost across countries, delivery strategies, vaccination sites, and phases of the rollout were primarily driven by variations in delivery volume, and operational and financial constraints.

Our study is the first to analyze the cost of COVID-19 vaccine delivery across phases of the introduction, and we found that scale and efficiencies had a large impact on the delivery cost. During the initial period, the pressure to quickly deliver small batches of vaccines to a small priority population caused inefficiencies that increased delivery costs. When supply constraints eased, and eligibility was extended to the general population, the unit cost of delivery decreased significantly. In Mozambique and Uganda, this even meant that daily delivery costs per site were lower when program delivered at scale, despite the much larger volume delivered.

Our results show that targeting a small priority population—as is again the case for many COVID-19 vaccination programs and other adult vaccination programs—can come at a high delivery cost per person reached, and for such programs, integrating delivery with other primary health care platforms is key to achieve efficiencies.

We also found evidence of economies of scale across vaccination sites, delivery modalities—with campaigns generally being less costly per dose compared to continuous delivery—and across countries. Bangladesh, Mozambique, and Vietnam all managed to reach relatively high daily delivery volumes, driving the unit cost of delivery down. DRC struggled with severe vaccine hesitancy and operational bottlenecks, resulting in a very high unit cost of delivery.

In the Philippines, we found a higher financial cost per dose despite the relatively high delivery volumes, driven by the mass hiring of additional health workers for their COVID-19 vaccination program. The Philippines is usually an exporter of health workers, but during the pandemic the country barred health workers from leaving to work abroad, creating a surplus that was leveraged for the COVID-19 response [21]. This was exceptional, and all other countries reported critical shortages, as they were only able to mobilize limited additional support through redeployment and volunteers.

We compared our results to the existing literature on COVID-19 vaccine delivery costs, and found eight country studies and one multi-country model [22]. Only two studies, respectively from Nigeria (financial cost $0·84) and Laos PDR (financial cost $0·79), found delivery costs similar to ours [15,23]. Most country studies found higher unit costs, including studies in Kenya (financial cost $1·65-$2·14, economic cost $3·67-$6·11, assuming 30%−100% coverage), Ghana ($2·2-$2·3), South Africa (financial cost $3·84, economic cost $10·38), Somalia ($5), remote Sierra Leone (economic cost $33), and Botswana (fiscal cost $19·12, economic cost $33·18) [9–11,13,14,17]. High costs for newly hired health workers (South Africa, Botswana), and greater resource needs in the specific settings assessed (Sierra Leone) explain some of the differences. Additionally, several studies relied on past practices or top-down assumptions which may have not been reflective of actual resource use at implementation level (Somalia, South Africa, Botswana, Kenya). The multi-country model estimated higher delivery costs for five of the seven countries in our study, as its aspirational assumptions were meant for global fund raising, and did not always reflect the resource-constrained realities [12].

In general, we found that the COVID-19 vaccination programs received very limited additional funding during the study periods for operational costs, cold chain expansions, and vehicles, which explains why the financial cost per dose found in our study is relatively low compared with existing publications on immunization delivery costs. Despite the tremendous vaccine volumes that needed to be delivered, and global commitments to support equitable access, we found severe resource constraints at implementation level, due to funding shortages and disbursement delays. This primarily affected transport and incentives given to health workers, who reported contributing their own resources to support vaccination activities.

Political prioritization, health workers’ extraordinary commitment, and volunteers were highlighted as key success factors to achieving high coverage. However, it is important to interpret these achievements—reaching high coverage at low delivery cost—within the emergency setting that the COVID-19 pandemic presented.

Our findings have important implications for planning and financing future pandemic responses. The low financial cost per dose observed in this study reflects actual resource use under extraordinary circumstances should not be interpreted as evidence that delivering vaccines at scale during a pandemic requires few financial resources. Instead, these low costs primarily reflect severe resource constraints and delays in disbursement, which resulted in insufficient funding for transport, cold chain, and incentives for health workers. While unprecedented political prioritization, the large-scale support of volunteers, and the extraordinary commitment of health workers enabled high coverage during the COVID-19 rollout, a reliance on underfunded health system and a large-scale redeployment of an already under-resourced health workforce is not sustainable. Therefore, preparing for future pandemics should entail timely investments in strengthening the routine delivery infrastructure, expanding the existing health workforce, and establishing flexible financing mechanisms, to ensure health systems can respond to emerging crises without disrupting the delivery of other essential health services.

Our study presents several limitations. First, the study sample was small, and sites were purposively selected due to challenges in obtaining a complete and accurate population of sites delivering COVID-19 vaccines. While efforts were made to capture diverse settings, findings may not be nationally representative. Second, our results only reflect the specific time periods observed, while COVID-19 programs changed significantly over time. Third, some non-health implementers and partners refused to share cost data, though as we included data from all major funders, we do not expect this to alter the interpretation of our findings. Finally, our analysis relied on reports from health workers which may have been subject to recall bias, and on data records whose accuracy could not always be validated, which may have introduced error in our estimates.

## Conclusion

In conclusion, our study fills a critical evidence gap on the cost of the COVID-19 vaccine roll-out in LMICs. Our findings represent actual country practices for rapidly vaccinating a large target population, in resource-constrained emergency settings. This is the first study to describe how costs changed as supply constraints eased and the target population evolved, as well as how costs differed across sites, delivery modalities, and countries. Our results can help to improve pandemic preparedness, and inform planning and budgeting for new vaccine roll-outs, as well existing vaccination programs.

## Supporting information

S1 Table Definition of resource types. (DOCX)

S2 Table Definition of program activities. (DOCX)

S3 Table Study period, by country. (DOCX)

S4 Table Imputation of missing data. (DOCX)

S5 Table Allocation rules for shared resources. (DOCX)

S6 Table Financial cost per dose in 2022 USD, by program activity. (DOCX)

S7 Table Economic cost per dose in 2022 USD, by program activity. (DOCX)

S8 Table Economic cost per dose in 2022 USD and doses delivered per site per day, by delivery strategy or delivery site. (DOCX)

S9 Table Cost per dose in 2022 USD (and % of economic costs per dose) and doses delivered per site per day, for different delivery modalities. (DOCX)
