# Supplementary material for: The cost and cost drivers of delivering COVID-19 vaccines in low- and middle-income countries: a bottom-up costing study of rollouts in seven countries
Source: PLoS One. 2026 Feb 2;21(2):e0341964. doi: 10.1371/journal.pone.0341964 (PMC12863507; doi:10.1371/journal.pone.0341964)
Supplement: S1 Table — (DOCX) [file pone.0341964.s001.docx]

**S1 Table. Definition of resource types.**

| **Resource type** | **Definition** |
| --- | --- |
| **Financial costs** | |
| **Labor – newly hired health staff** | Salary paid to new employees hired specifically for the COVID-19 vaccination program. |
| **Per diem & incentives** | Daily allowances, subsidies, travel allowances, honorarium, financial and in-kind (e.g. refreshments) incentives paid to regular employees and volunteers for participation in activities related to the COVID-19 vaccination program. |
| **Vaccine administration supplies** | Cost of vaccine injection and safety supplies, safety boxes, other supplies and personal protective equipment used for administration of COVID-19 vaccines. |
| **Transport and fuel** | Cost of bus fare, plane travel, boat travel/hire vehicle hire and fuel for COVID-19 vaccination program activities that required travel (supervision, training, vaccine collection, distribution, etc.) Fuel costs for the waste disposal incinerator used for COVID-19 vaccine-related waste management. |
| **Other financial costs** | Cold chain repairs and energy costs (cost of cold chain equipment repairs carried out specifically for the COVID-19 vaccination program during the study period and cost for the cold equipment is the storage room energy bill, if available); communication costs (costs related to purchasing airtime and mobile data for the purpose of the COVID-19 vaccination program.); IEC and printing costs (cost of printing immunization cards, training materials, radio and tv appearances and other IEC materials that are related to the COVID-19 vaccination program.); vehicle maintenance (cost of vehicle maintenance carried out specifically for the COVID-19 vaccination program during the study period); workshops and meeting costs (cost incurred specifically for COVID-19 vaccination workshops and meetings (e.g. venue hire, refreshments, mobile data for virtual meetings); stationery and other supplies (cost of stationery and other supplies used for the COVID-19 vaccination program); other financial costs related to the COVID-19 vaccination program (e.g. newly purchased laptops procured exclusively for the COVID-19 vaccination program). |
| **New equipment** | Newly purchased cold chain equipment, vehicles, incinerators and other equipment that were procured specifically for the COVID-19 vaccination effort and used during the study period. |
| **Opportunity costs** | |
| **Labor – existing health staff** | Share of the salary paid to health workers and government employees proportional to the time they spent working on activities related to the COVID-19 vaccination program, and based on their salary grade. |
| **Unpaid labor** | Value of voluntary work (performed by medical students, local youth, retired health staff, community representatives etc.) for vaccination team members who do not receive a regular salary. This cost was calculated based on the working hours of each volunteer and valued at a minimum wage for the public sector. For the DRC, also includes the value of labor of regular health staff that did not receive a salary, calculated based on hours worked on the hourly salary that they should have received based on their salary grade. |
| **Other opportunity costs** | Depreciation costs of already owned vehicles, cold chain equipment, and incinerators and costs related to routine cold chain repairs. |
