# Supplementary material for: The cost and cost drivers of delivering COVID-19 vaccines in low- and middle-income countries: a bottom-up costing study of rollouts in seven countries
Source: PLoS One. 2026 Feb 2;21(2):e0341964. doi: 10.1371/journal.pone.0341964 (PMC12863507; doi:10.1371/journal.pone.0341964)
Supplement: S2 Table — (DOCX) [file pone.0341964.s002.docx]

**S2 Table. Definition of program activities.**

| **Program activity** | **Definition** |
| --- | --- |
| **Program management** | COVID-19 vaccination program management, including development of guidelines, program meetings, development of vaccination implementation plans, budgeting for the program. |
| **Vaccine collection, distribution and storage** | Storing vaccines in national level cold storages, distributing vaccines from the national level to intermediary and lower administrative levels and down to health facilities and other vaccination sites. |
| **Cold chain maintenance** | Maintaining and repairing the cold chain for the purpose of the COVID-19 vaccine roll-out. |
| **Training** | Attending and/or providing COVID-19 vaccination-related training, including topics such as administering vaccines, storage and logistics, record keeping, pharmacovigilance, social mobilization, planning, supervision, etc. |
| **Social mobilization and advocacy** | Mobilizing and sensitizing the community and households, conducting social mobilization events, and advocating for COVID-19 vaccination, including developing and distributing social mobilization and advocacy materials, via mass media, social media, leaflets, etc. |
| **Supervision** | Supervising subordinate or peer health or community workers. |
| **Service delivery** | Administering the vaccine to beneficiaries at the vaccination sites, including preparation and cleaning up before and after the vaccination event. |
| **Waste management** | Time and resources spent on disposing sharps and infectious non-sharp wastes. |
| **Adverse Effects Following Immunization (AEFI) monitoring & management** | Monitoring, managing, following up and reporting on post-vaccination adverse effects following COVID-19 vaccine administration. |
| **Record-keeping, HMIS, monitoring and evaluation** | Data entry and analysis, reporting, monitoring. |
