# Supplementary material for: The cost and cost drivers of delivering COVID-19 vaccines in low- and middle-income countries: a bottom-up costing study of rollouts in seven countries
Source: PLoS One. 2026 Feb 2;21(2):e0341964. doi: 10.1371/journal.pone.0341964 (PMC12863507; doi:10.1371/journal.pone.0341964)
Supplement: S3 Table — (DOCX) [file pone.0341964.s003.docx]

**S3 Table. Study period, by country.**

|  | **Period for which data was collected** | | **Disaggregated analyses conducted** | | |
| --- | --- | --- | --- | --- | --- |
|  | **One-off investments** | **Recurrent costs** | **Introduction phase** (initial rollout targeting priority groups vs scaled up phase targeting general population) | **Delivery modality** (continuous vs campaigns) | **Delivery strategy or type of site** |
| **Bangladesh** | Dec-20 to Oct-22 | Apr-22 to Jun-22 (continuous)  Sept-22 to Oct-22 (campaigns) | No, data not collected for initial rollout | Yes | Yes |
| **Côte d’Ivoire** | Feb-21 to May-22 | Mar-22 to May-22 (continuous) | No, due to data limitations | No, due to data limitations | No, due to data limitations |
| **The DRC** | Mar-21 to Jun-22 | Nov-21, and Apr-22 to May-22 (campaigns)  Jun-22 or latest available month (continuous) | No, data not collected for initial rollout | Yes | No, due to data limitations |
| **Mozambique** | Feb-21 to Feb-22 | Mar-21 to Apr-21 (initial rollout)  Dec-21 to Feb-22 (scaled up) | Yes | Not applicable | No, due to data limitations |
| **The Philippines** | Feb -21 to Jul-22 | Nov-21 to Dec-21 (campaigns)  May-22 to Jul-22 (continuous) | No, data not collected for initial rollout | Yes | Yes |
| **Uganda (Kampala)** | Feb -21 to Dec-21 | Jan-21 to Jun-21 (initial rollout) Jul-21 to Dec-21 (scaled up) | Yes | Not applicable | No, due to data limitations |
| **Vietnam** | Feb -21 to Dec-21 | Mar-21 to Jun-21 (initial rollout)  Jul-21 to Dec-21 (scaled up) | Yes | Not applicable | Yes |
