# Supplementary material for: The cost and cost drivers of delivering COVID-19 vaccines in low- and middle-income countries: a bottom-up costing study of rollouts in seven countries
Source: PLoS One. 2026 Feb 2;21(2):e0341964. doi: 10.1371/journal.pone.0341964 (PMC12863507; doi:10.1371/journal.pone.0341964)
Supplement: S4 Table — (DOCX) [file pone.0341964.s004.docx]

**S4 Table. Imputation of missing data.**

| **Missing data** | **Imputation method** |
| --- | --- |
| **Quantity used for vaccine administration and safety supplies** | Imputed based on the average quantity used during other periods at the same site, or if quantity was missing for all periods, based on quantity used at other vaccination sites, per COVID-19 vaccine dose delivered. |
| **Transport fuel costs** | - If distance travelled was provided, fuel costs were imputed using local assumptions of liters of fuel used per km (adjusted with a modifier for traffic congestion, if relevant) and based on local fuel prices. - If distance travelled was also not available, fuel costs were imputed based on the average fuel cost per day of vehicle use at other sites, calculated only including the same type of vehicle. |
| **Waste management costs** | Imputed based on the average cost per dose at all other sites. |
| **Salary for health staff** | Imputed based on the average salary for staff of the same cadre of at the same study site, and if not available, based on the average salary for staff of the same cadre at other sites. |
| **Salary for staff at partners’ organizations** | Imputed based on the reported job title and a dataset of salary information from partner organizations pooled from several immunization costing studies conducted by the study team (in Sierra Leone, Nigeria, Mozambique and Vietnam). |
| **Doses delivered at vaccination site** | Imputed this based on the average doses delivered at comparable vaccination sites. Comparability was established based on vaccination site staffing levels and number of COVID-19 vaccination boots, or number of childhood routine vaccine doses delivered during the study period. |
| **Staff hours** | Imputed based on staff hours for similar cadres at the same site. |
| **Procurement price of CCE or vehicle** | Where the procurement price of a newly purchased cold chain equipment item or vehicle was not reported, we used the current market price of that item, based on its make and model. |
