# Supplementary material for: The cost and cost drivers of delivering COVID-19 vaccines in low- and middle-income countries: a bottom-up costing study of rollouts in seven countries
Source: PLoS One. 2026 Feb 2;21(2):e0341964. doi: 10.1371/journal.pone.0341964 (PMC12863507; doi:10.1371/journal.pone.0341964)
Supplement: S5 Table — (DOCX) [file pone.0341964.s005.docx]

**S5 Table. Allocation rules for shared resources.**

Resources shared between the health system and the COVID-19 vaccine program or across multiple COVID-19 program activities implementation periods or delivery strategies (e.g. labor of health staff), were always allocated based on use as reported by health staff or based on written records. When this was not possible, the following general rules were applied:

| **Shared costs** | **Allocation rules** |
| --- | --- |
| **Resources shared across the health system** | |
| - Cold chain equipment costs - Cold chain energy costs - Waste disposal fuel costs | % of space occupied by COVID-19 vaccines in cold chain equipment or % COVID-19 vaccine doses delivered at that site |
| - Cold chain maintenance costs | % of space occupied by COVID-19 vaccines in cold chain equipment or % of cold chain equipment items that were maintained used for COVID-19 vaccines |
| - Vehicle maintenance costs | % vehicles at that site used for the COVID-19 vaccine program |
| - General printing costs - General fuel costs | % staff time spent on COVID-19 vaccination program vs. total working time |
| **Resources shared across multiple COVID-19 program activities** | |
| - Lump sum per diem, incentives or allowances | % of staff time spent on each program activity |
| - Fuel for transport - Vehicle maintenance costs | % of staff time spent on program activities that required transportation (including supervision, training, distribution, and vaccine collection) |
| **Resources shared across COVID-19 program implementation periods or delivery strategies** | |
| - Printing costs for registers - Printing cost for vaccination cards - Vaccine distribution third party contracts - Waste management third party contracts - Cold chain equipment costs - Vehicles costs - Cold chain and vehicles maintenance costs | % doses delivered in each period or through each delivery strategy |
| - Labor and other costs related to preparatory activities (e.g. training, planning), assuming a useful life of 1 year | % of time in each delivery period |
