# Supplementary material for: The cost and cost drivers of delivering COVID-19 vaccines in low- and middle-income countries: a bottom-up costing study of rollouts in seven countries
Source: PLoS One. 2026 Feb 2;21(2):e0341964. doi: 10.1371/journal.pone.0341964 (PMC12863507; doi:10.1371/journal.pone.0341964)
Supplement: S6 Table — (DOCX) [file pone.0341964.s006.docx]

**S6 Table. Financial cost per dose in 2022 USD, by program activity.**

|  | **Vietnam** | **Bangladesh** | **The Philippines** | **Uganda (Kampala)** | **Mozambique** | **Cote d'Ivoire** | **The DRC** |
| --- | --- | --- | --- | --- | --- | --- | --- |
| Cold chain maintenance | ·· | 0·00 | 0·03 | 0·00 | 0·00 | 0·01 | 0·03 |
| Record keeping, HMIS, M&E | 0·00 | 0·01 | 0·23 | 0·09 | 0·08 | 0·05 | 0·07 |
| Social mobilization and advocacy | 0·00 | 0·03 | 0·04 | 0·06 | 0·04 | 0·04 | 0·14 |
| Supervision | 0·00 | 0·00 | 0·06 | 0·06 | 0·03 | 0·02 | 0·14 |
| Training | 0·01 | 0·01 | 0·01 | 0·05 | 0·00 | 0·00 | 0·03 |
| Vaccine administration | 0·54 | 0·05 | 0·95 | 0·29 | 0·26 | 0·30 | 1·18 |
| Vaccine collection, distribution and storage | 0·04 | 0·03 | 0·30 | 0·04 | 0·03 | 0·11 | 0·11 |
| Waste management | 0·01 | 0·01 | 0·03 | 0·08 | 0·04 | 0·11 | 0·04 |
| AEFI monitoring and management | 0·00 | 0·00 | 0·31 | 0·01 | 0·00 | 0·00 | 0·01 |
| Program management | 0·00 | 0·00 | 0·04 | 0·13 | 0·01 | 0·02 | 0·13 |
| Crowd controlling & client management | ·· | 0·15 | ·· | ·· | ·· | ·· | ·· |
| Other activities | 0·00 | ·· | ·· | ·· | 0·00 | 0·01 | 0·30 |
| **All activities** | **0·60** | **0·29** | **1·99** | **0·82** | **0·50** | **0·67** | **2·18** |
