# Supplementary material for: The cost and cost drivers of delivering COVID-19 vaccines in low- and middle-income countries: a bottom-up costing study of rollouts in seven countries
Source: PLoS One. 2026 Feb 2;21(2):e0341964. doi: 10.1371/journal.pone.0341964 (PMC12863507; doi:10.1371/journal.pone.0341964)
Supplement: S7 Table — (DOCX) [file pone.0341964.s007.docx]

**S7 Table. Economic cost per dose in 2022 USD, by program activity.**

|  | **Vietnam** | **Bangladesh** | **The Philippines** | **Uganda (Kampala)** | **Mozambique** | **Cote d'Ivoire** | **The DRC** |
| --- | --- | --- | --- | --- | --- | --- | --- |
| Cold chain maintenance | 0·00 | 0·01 | 0·05 | 0·10 | 0·02 | 0·03 | 0·13 |
| Record keeping, HMIS, M&E | 0·13 | 0·04 | 0·46 | 0·53 | 0·16 | 0·26 | 1·15 |
| Social mobilization and advocacy | 0·05 | 0·05 | 0·12 | 0·20 | 0·19 | 0·35 | 0·72 |
| Supervision | 0·10 | 0·06 | 0·18 | 0·16 | 0·17 | 0·13 | 0·88 |
| Training | 0·03 | 0·02 | 0·01 | 0·15 | 0·01 | 0·13 | 0·28 |
| Vaccine administration | 1·24 | 0·48 | 1·66 | 0·59 | 0·36 | 1·55 | 2·43 |
| Vaccine collection, distribution and storage | 0·07 | 0·07 | 0·40 | 0·15 | 0·09 | 0·19 | 0·40 |
| Waste management | 0·02 | 0·01 | 0·04 | 0·10 | 0·06 | 0·13 | 0·71 |
| AEFI monitoring and management | 0·00 | 0·01 | 0·42 | 0·10 | 0·03 | 0·06 | 0·60 |
| Program management | 0·06 | 0·05 | 0·16 | 0·36 | 0·05 | 0·27 | 1·83 |
| Crowd controlling & client management | ·· | 0·23 | ·· | ·· | ·· | ·· | ·· |
| Other activities | 0·07 | ·· | ·· | ·· | 0·00 | 0·06 | 0·38 |
| **All activities** | **1·78** | **1·05** | **3·50** | **2·43** | **1·14** | **3·16** | **9·50** |
