# Supplementary material for: The cost and cost drivers of delivering COVID-19 vaccines in low- and middle-income countries: a bottom-up costing study of rollouts in seven countries
Source: PLoS One. 2026 Feb 2;21(2):e0341964. doi: 10.1371/journal.pone.0341964 (PMC12863507; doi:10.1371/journal.pone.0341964)
Supplement: S8 Table — (DOCX) [file pone.0341964.s008.docx]

**S8 Table. Economic cost per dose in 2022 USD and doses delivered per site per day, by delivery strategy or delivery site.**

| **Type of delivery site** | **Number of sites** | **Dose delivered per day/site** | **Cost per dose (2022 USD)** | |
| --- | --- | --- | --- | --- |
|  |  |  | **Financial** | **Economic** |
| **Vietnam** | | | | |
| **Fixed sites** | 14 | 174 | 0·67 | 1·67 |
| **Temporary sites** | 12 | 350 | 0·58 | 1·82 |
| **Bangladesh** | | | | |
| **MOH hospitals** | 14 | 717 | 0·29 | 0·99 |
| **Other government hospitals** | 5 | 271 | 0·36 | 2.15 |
| **EPI centres** | 7 | 201 | 0·44 | 1·83 |
| **Schools** | 6 | 983 | 0·27 | 0·74 |
| **Temporary mass sites** | 6 | 320 | 0·33 | 1·08 |
| **The Philippines – campaign delivery** | | | | |
| **Rural health units** | 14 | 420 | 0·60 | 2·72 |
| **Private clinics** | 1 | 567 | 1·84 | 2·15 |
| **Temporary mall sites** | 4 | 883 | 1·13 | 1·76 |
| **City health offices** | 5 | 1060 | 1·56 | 2·11 |
| **The Philippines – continuous delivery** | | | | |
| **Rural health units** | 14 | 18 | 2·55 | 12·64 |
| **Private clinics** | 4 | 188 | 3·51 | 3·87 |
| **Temporary mall sites** | 4 | 258 | 2·03 | 4·06 |
| **City health offices** | 4 | 916 | 2·32 | 3·82 |
