# Supplementary material for: The cost and cost drivers of delivering COVID-19 vaccines in low- and middle-income countries: a bottom-up costing study of rollouts in seven countries
Source: PLoS One. 2026 Feb 2;21(2):e0341964. doi: 10.1371/journal.pone.0341964 (PMC12863507; doi:10.1371/journal.pone.0341964)
Supplement: S9 Table — (DOCX) [file pone.0341964.s009.docx]

**S9 Table. Cost per dose in 2022 USD (and % of economic costs per dose) and doses delivered per site per day, for different delivery modalities.**

|  | **Bangladesh** | | **Philippines** | | **DRC** | |
| --- | --- | --- | --- | --- | --- | --- |
| **Delivery modality** | **Continuous** | **Campaign** | **Continuous** | **Campaign** | **Continuous** | **Campaign** |
| **Number of sites** | 26 | 6 | 26 | 24 | 26 | 26 |
| **Dose delivered per day/site** | 493 | 320 | 161 | 598 | 18 | 52 |
| **% of sites with diem*** | 92% | 100% | 42% | 71% | 23% | 35% |
| **Average daily diem*****per staff (at sites where they were provided) in 2022 USD** | 2·95 | 5·24 | 1·90 | 3·54 | 4·78 | 10·46 |
| **Financial costs in 2022 USD** (% of economic costs per dose) | | | | | | |
| Labor - newly hired health staff | 0·01 (1%) | 0·01 (1%) | 1·34 (31%) | 0·68 (31%) | 0·11 (1%) | 0·01 (0%) |
| Per diem & incentives* | 0·20 (18%) | 0·19 (17%) | 0·04 (1%) | 0·31 (14%) | 1·18 (6%) | 0·64 (10%) |
| Vaccine admin. supplies | 0·04 (4%) | 0·05 (5%) | 0·27 (6%) | 0·16 (7%) | 0·65 (3%) | 0·45 (7%) |
| Transport and fuel | 0·02 (2%) | 0·03 (2%) | 0·07 (2%) | 0·01 (0%) | 0·29 (1%) | 0·19 (3%) |
| Other financial costs^†^ | 0·03 (3%) | 0·05 (5%) | 0·55 (13%) | 0·25 (12%) | 0·87 (4%) | 0·61 (10%) |
| New equipment | 0·01 (1%) | 0·00 (0%) | 0·07 (2%) | 0·01 (0%) | 0·00 (0%) | 0·00 (0%) |
| **Total financial cost per dose** | **0·30 (28%)** | **0·33 (31%)** | **2·35 (55%)** | **1·41 (65%)** | **3·12 (16%)** | **1·91 (31%)** |
| **Opportunity costs in 2022 USD** (% of economic costs per dose) | | | | | | |
| Labor for existing health staff | 0·68 (64%) | 0·66 (61%) | 1·84 (43%) | 0·65 (30%) | 9·24 (47%) | 2·37 (39%) |
| Unpaid labor | 0·05 (5%) | 0·07 (6%) | ·· | 0·08 (4%) | 6·22 (32%) | 1·63 (27%) |
| Other opportunity costs | 0·02 (2%) | 0·02 (2%) | 0·10 (2%) | 0·02 (1%) | 0·99 (5%) | 0·19 (3%) |
| **Total opportunity cost per dose** | **0·76 (72%)** | **0·75 (70%)** | **1·95 (45%)** | **0·75 (35%)** | **16·45 (84%)** | **4·19 (69%)** |
| **Economic cost per dose** | **1·06** | **1·08** | **4·29** | **2·16** | **19·56** | **6·10** |

*Also includes refreshments for vaccination team members and allowances for volunteers; ^†^ For DRC, other costs are mostly made up of donor contributions spent on unknown resource types
